# Unraveling the potential: mRNA therapeutics in oncology

**DOI:** 10.3389/fonc.2025.1643444

**Published:** 2025-08-13

**Authors:** Karol Gawalski, Weronika Przybyszewska, Jaromir Hunia, Alicja Gawalska, Aleksandra Rymarz

**Affiliations:** ^1^ Laboratory of Experimental Medicine, Medical University of Warsaw, Warsaw, Poland; ^2^ Doctoral School, Medical University of Warsaw, Warsaw, Poland; ^3^ Department of Nephrology, Dialysis and Internal Medicine, Medical University of Warsaw, Warsaw, Poland; ^4^ Drug Discovery and Early Development Department, Adamed Pharma S.A., Czosnów, Poland

**Keywords:** mRNA, vaccines, therapeutic mRNA, tumor-specific antigens, tumor-associated antigens, cancer, clinical trials

## Abstract

Messenger ribonucleic acid (mRNA) technology is a promising platform for cancer immunotherapy. Unlike traditional vaccines that prevent infectious diseases, mRNA’s role in oncology is to stimulate or enhance the immune response against tumor antigens. This review provides an overview of mRNA’s historical development, from its discovery in 1961 to recent clinical trials and Nobel Prize-winning breakthroughs. Therapeutic mRNA flexibility allows the alteration of diverse tumor antigens. Key targets include tumor-associated antigens, which are present on both tumor cells and some healthy cells, as well as tumor-specific antigens unique to cancer cells, such as antiviral antigens and neoantigens arising from tumor mutations. Various approaches to protect mRNA from degradation, including protamine-complexed mRNA, lipoplexes, and lipid nanoparticles, as well as several administration routes, are currently being tested in clinical trials. They are focused on malignancies like melanoma, non-small cell lung cancer, prostate cancer, or pancreatic ductal adenocarcinoma, one of the most challenging cancers. While many trials are in early phases, some have advanced to phase 3 and have shown promising results in both safety and efficacy. However, due to the complexity and heterogeneity of tumors, even among patients presenting the same subgroup of neoplasm, fully universal mRNA-based cancer vaccine seems to be elusive. Personalized mRNA cancer vaccines targeting neoantigens unique to an individual’s tumor have gained traction as a feasible and promising solution. Technological advances in bioinformatics, AI, and machine learning now allow for more accurate identification of immunogenic neoepitopes. The combination this type of therapy with other treatment such as immune checkpoint inhibitors may become one of new solutions in oncology.

## 1 Introduction

Over the last couple of decades, messenger ribonucleic acid (mRNA) has demonstrated to be a promising platform for therapeutic applications. The coronavirus disease 2019 (COVID-19) pandemic was a global disaster that has challenged healthcare systems and economies worldwide. It also has left a lasting impact on the nowadays world. For the first time, we experienced how fast mRNA vaccines can be designed, produced, and registered to successfully induce a protective immune response. In October and November 2020, vaccine industry companies published the initial results of phase 1/2b clinical trials for anti-COVID-19 mRNA vaccines, less than a year after severe acute respiratory syndrome coronavirus 2 (SARS-CoV-2) emergence (1, 2). These data proved the effectiveness of the mRNA vaccines used for the protection of new world-wrecking threat. Anti-COVID-19 mRNA vaccines have achieved success due to efforts of thousands of scientists that have been working on understanding and improving mRNA technology for the last several decades. The culmination, but also a new impetus, was the Nobel Prize in Physiology or Medicine in 2023 for Katalin Karikó and Drew Weissman, which confirmed the weights of breakthrough discoveries in mRNA area (3).

**Figure 1:**
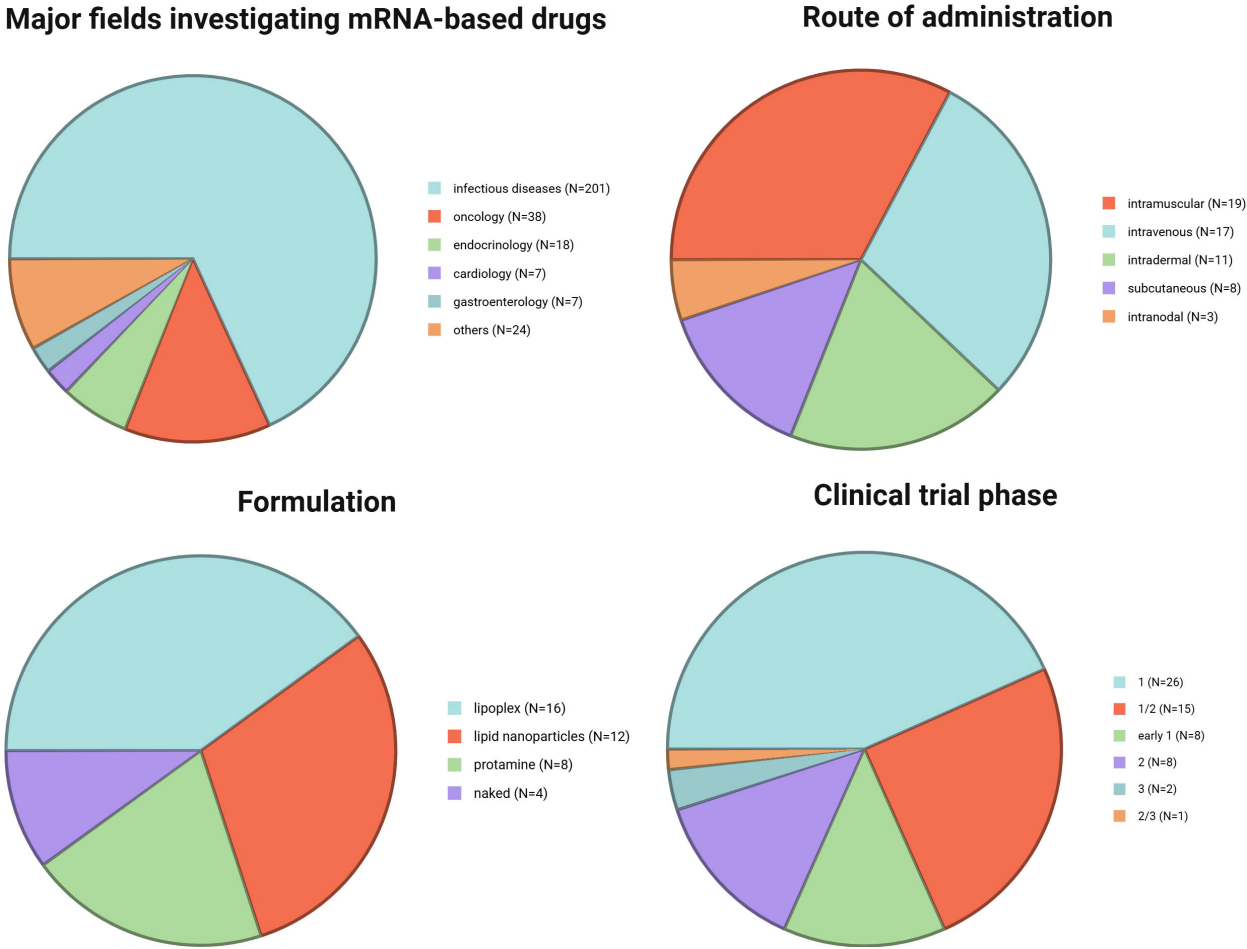
Major fields investigating mRNA-based drugs [data based on analysis with Clarivate’s Cortellis Competitive Intelligence Database presented and discussed in (4)] and summary of the analyzed clinical trials: route of administration, formulation, and trial phase. Created in BioRender. Gawalska, A (2025) https://BioRender.com/b7lzjsf.

**Figure 2:**
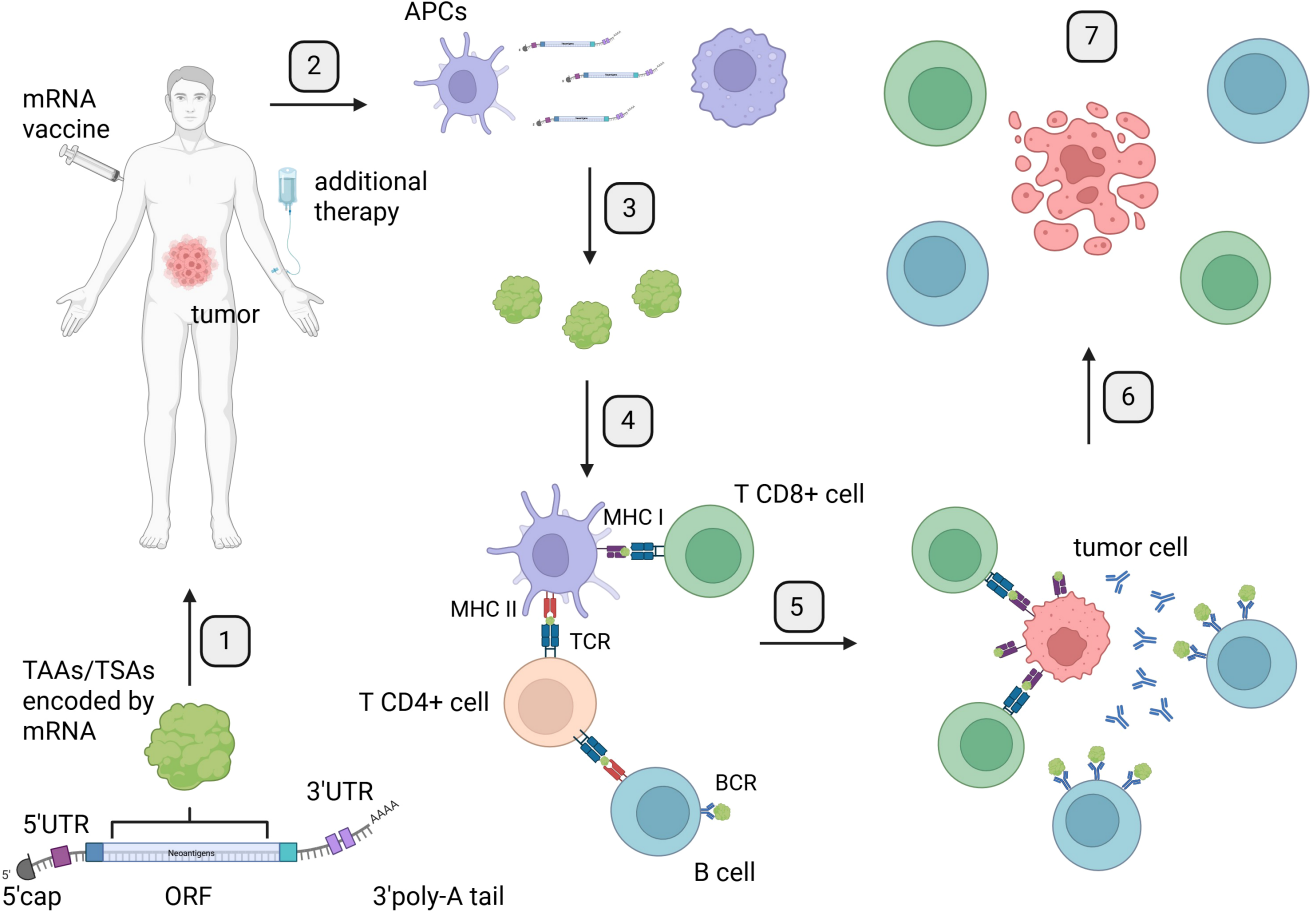
Mechanism of mRNA therapeutic action: from vaccine preparation to induction of immune response and tumor cell elimination. Created in BioRender. Gawalska, A (2025) https://BioRender.com/rt44g17. 1. Identification and selection of TAAs or TSAs encoded by the Open Reading Frame (ORF) of mRNA for the development of mRNA therapeutics. 2. Administration of mRNA vaccines to patients via a chosen route, along with additional therapy such as ICIs. 3. Internalization of mRNA by antigen-presenting cells (APCs) through endocytosis. 4. Translation of mRNA and processing of proteins by APCs for presentation to T cells by the major histocompatibility complex. 5. Presentation of antigens encoded by mRNA to T cells by APCs and B cells, leading to activation of the immune response. 6. Activation of immune response by B and T cells targeting tumor cells. 7. Induction of tumor cells death by an effective immune response from B and T cells. APCs, antigen-presenting cells; BCR, B-cell receptor; TCR, T-cell receptor; MHC I, major histocompatibility complex class I; MHC II, major histocompatibility complex class II.

mRNA therapeutics due to their flexibility can be potentially used in medicine (**Figure 1**). The majority of studies try to implement mRNA products against microbes, which caused infectious diseases. Oncology is the second most popular field where clinical trials evaluate usefulness of mRNA products. Attempts to use vaccines in cancer treatment present significantly different goals than in protection against microbes. In general, vaccination leads to the development of antigen-specific B and T cells, which can recognize pathogen-derived antigens and protect us against infectious disease. In oncology, vaccines are applied after cancer diagnosis. The main concept of anticancer vaccines is to induce and/or strengthen the immune response targeting tumor-specific (TSAs) or tumor-associated (TAAs) antigens. The goal of cancer immunotherapy is to cure the patient from the tumor (**Figure 2**).

In this review article, we aim to provide a brief overview of the background of mRNA use in clinical trials within the field of oncology. We will also discuss the status of ongoing clinical trials and highlight the latest groundbreaking results in this area published over the past few years. In the end, we present also future perspectives and obstacles that mRNA technology is facing today.

## 2 From bench to bedside: a brief history of therapeutic mRNA development

### 2.1 Foundational discovery

The journey of mRNA therapeutics toward their use in clinical trials began with Brenner, Jacob, and Meselson’s description of mRNA particles in 1961 (5). Further insights into its structure, especially the 3′ poly-adenosine(A) tail (6) and the 5′ cap (7) led to two major advancements: the discovery of the enzyme required for ex vivo mRNA capping (8), and the development of a purification method (9).

### 2.2 Delivery innovations

These breakthroughs led to the first attempts to introduce mRNA into cells (10, 11). At this point, to further develop the mRNA technology, a scalable method for producing mRNA was required. Successful synthesis of *in vitro*-transcribed mRNA solved this challenge (12–14). Following effective protein expression from liposome-delivered mRNA (15, 16), Malone and colleagues applied this approach and observed successful translation after administering these formulations to human cells (17). Finally, researchers delivered mRNA into living organisms (18, 19). This established a foundation for investigating mRNA as a platform for inducing antigen-specific immune responses.

### 2.3 Immunological applications

The induction of the antiviral immune response after administration of mRNA encoding viral nucleoprotein (20) and expansion of virus-specific cytotoxic T lymphocytes (CTLs) (21) were breakthrough discoveries. These experiments demonstrated the potential of mRNA to elicit targeted immunity—a key requirement for effective antitumor therapies. This culminated in the presence of anti-carcinoembryonic antigen (CEA) antibodies (22) and anti-beta-galactosidase antibodies and CTLs (23) after mRNA administration, which supported the idea of delivering mRNA encoding tumor antigens.

### 2.4 Clinical translation

It was essential to develop lipid nanoparticles (LNPs) (24), which are one of the most employed ways to introduce mRNA into the human body in clinical trials today. Another breakthrough was the successful administration of mRNA into dendritic cells (DCs) and proved that their efficacy as a vaccine in a mouse model (25) inspired the idea for immunotherapy with DC-mRNA vaccines. Finally, Karikó and Weissman (26, 27) identified a major barrier to the clinical use of synthetic mRNA: its recognition by the innate immune system. Unmodified mRNA activates pattern recognition receptors (PRRs), triggering a strong interferon-mediated immune response. For the safe use of mRNA as a therapeutic, they developed a groundbreaking solution: chemical modification of uridine. Replacing uridine with pseudouridine allowed the mRNA to avoid detection by the immune system.

The entire journey of mRNA technology from its discovery through successive improvements and modifications culminated in its administration to patients leading to the induction of an immune response (28). The first clinical trial results, published in 2008, demonstrated the safety of injecting mRNA encoding tumor antigens (29). The treatment was immunogenic but did not result in therapeutic benefit in clinical settings. Since then, many researchers have focused on the application of mRNA in cancer immunotherapy, with a record number of studies in 2023 (4).

## 3 How mRNA therapeutics work—mRNA structure, formulations and route of administrations

### 3.1 mRNA as a therapeutic in clinic

**Figure 3:**
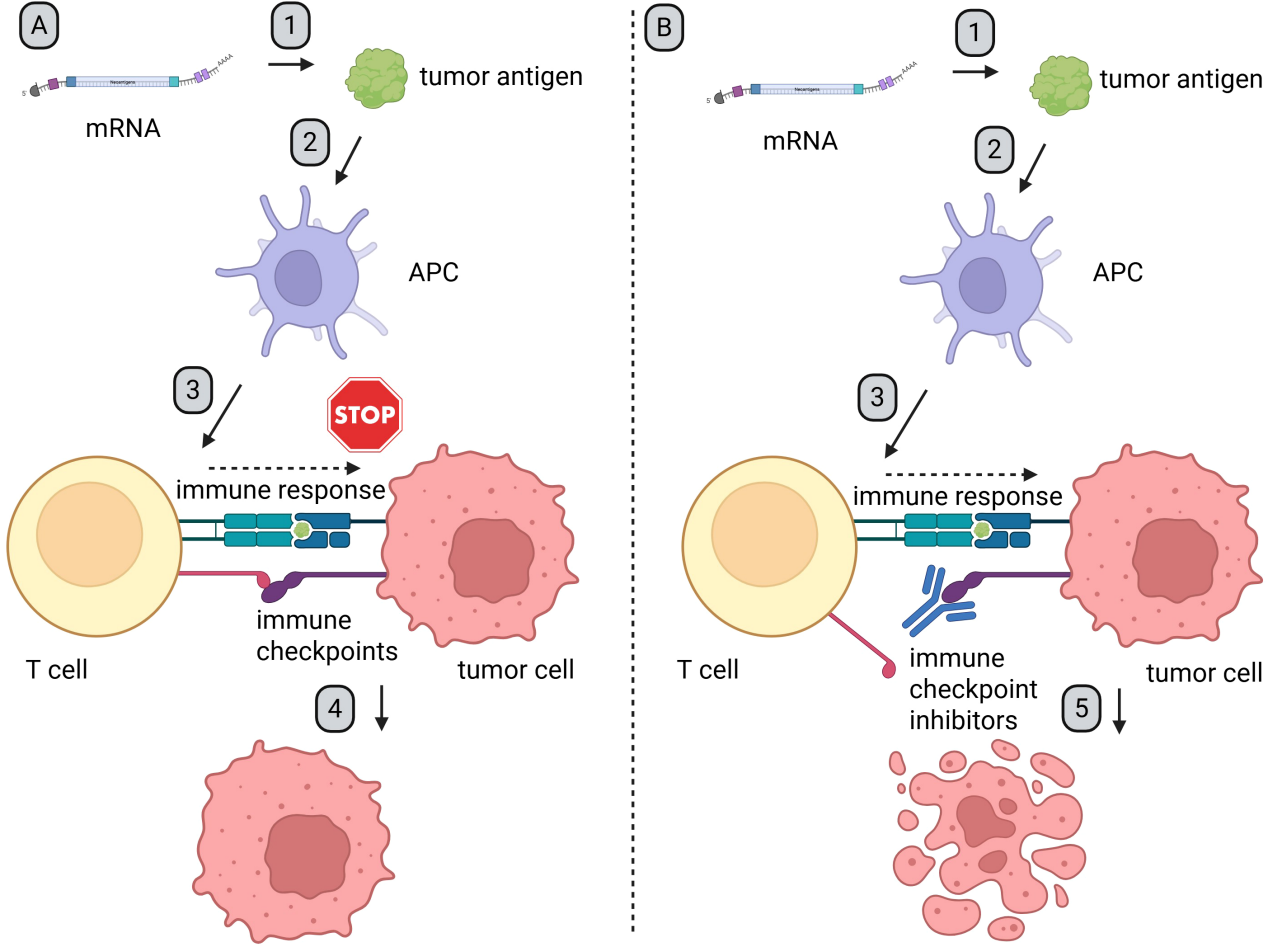
Mechanism of synergy between mRNA-based therapeutics and ICIs. Created in BioRender. Gawalska, A (2025) https://BioRender.com/zm73ph3. 1. After administration, APCs endocytosed mRNA particles, which leads to expression of the encoded antigen. 2. The antigens are processed and presented on MHC molecules. 3. Antigen presentation induces an immune response and activates both CD4^+^ and CD8^+^ T cells. 4. Tumor cells, which often overexpress ligands for immune checkpoint receptors, are able to evade immune surveillance and sustain proliferation (Figure A). 5. After administration of ICIs, checkpoint proteins and/or their ligands are blocked. This prevents tumor-induced immune suppression. As a result, T cell-mediated responses are restored, leading to an effective anti-tumor response (Figure B). APC, antigen-presenting cells; ICIs, immune checkpoint inhibitors.

Even though mRNA has been examined and modified for decades, its structure as a therapeutic remains similar to that found in our body. Therapeutic mRNA particles consist of a 5′cap, 5′ untranslated region (UTR), ORF—this part encodes final protein, 3′ UTR, and poly-A tail (**Figure 2**). Modifications in any of these elements play an important role in the enhancement of mRNA potential as a therapeutic agent (30). The utilization of therapeutic mRNA with distinct ORFs holds potential for inducing immune response against diverse tumors or even the same tumors with varying antigens on the surface of cancer cells. Additionally, the possibilities of changing ORFs are countless. This property makes mRNA a quick and flexible platform to induce an antigen-specific immune response in cancer patients and presents as a potential therapeutic agent with additional combined therapy like immune checkpoint inhibitors (ICIs) (**Figure 3**).

### 3.2 Targets for therapeutic mRNA in oncology

**Figure 4:**
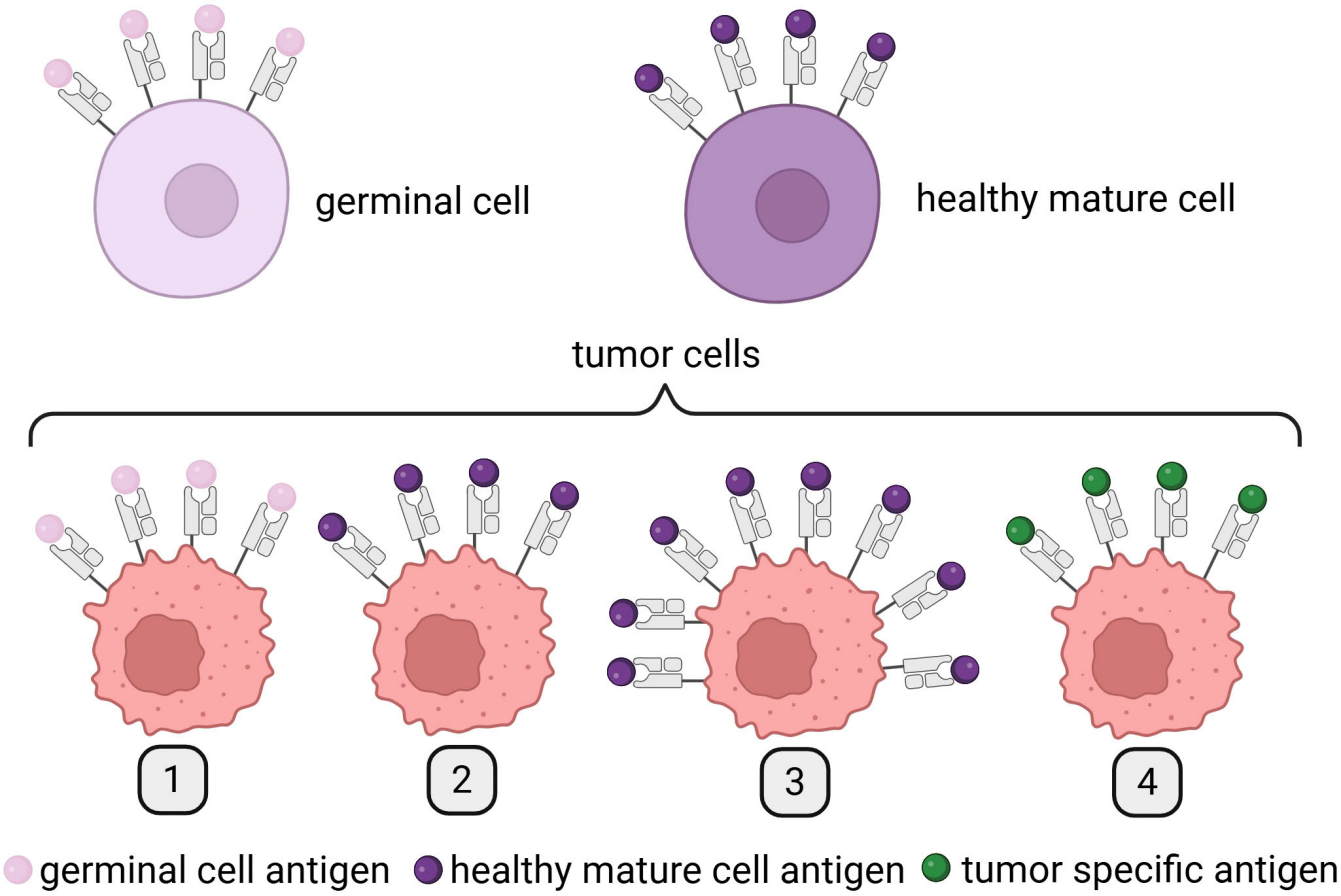
Differences between TAAs and TSAs. Created in BioRender. Gawalska, A. (2025) https://BioRender.com/ye0desp. TAAs include (1) antigens expressed on both germinal and tumor cells; (2) antigens expressed on tumor cells and the healthy tissue from which the tumor originates; and (3) antigens presented on healthy cells but overexpressed on tumor cells. In contrast, TSAs are (4) antigens exclusively expressed on tumor cells, arising as a result of tumor-specific mutations and/or viral oncogenesis. TAAs, tumor-associated antigens; TSAs, tumor-specific antigens, TSAs.

The antigens presented on the surface of cancer cells, which are the targets for immune response induced by therapeutic mRNA (**Figure 2**), generally belong to one of two primary categories: TAA or TSA (**Figure 4**).

TAAs can be divided into three different subgroups. Antigens from the first subgroup are presented on tumor cells and can be detected on germinal cells. The best example are melanoma-associated antigens (MAGEs) (31). The second subgroup of antigens is expressed on tumor cells and cells of a tissue from which cancer originated. In this group, we can find melanoma antigen recognized by T cells 1 (Melan-A/MART-1)—an antigen expressed on melanoma cells and healthy melanocytes (32, 33). Finally, antigens from the third subgroup are expressed in a healthy tissue and overexpressed on tumor cells such as human epidermal growth factor receptor 2 (Her-2/Neu) (34).

Today, we know dozens of TAAs present on the surface of tumor cells and healthy tissues; however, in the best-case scenario for the greatest efficacy of the treatment, antigens are only presented on cancer cells. This type of antigens is called TSAs. TSAs are divided into two groups: oncoviral antigens (caused by viral infection of cells) and neoantigens (as results of somatic mutations in cancer cells), the latter being the most promising option for personalized anticancer vaccines. At present, two different classifications of neoantigens exist: mutation-wise, which focuses on identification of mutated genes and the “immunogenic” that tries to find a link between the type of neoantigen and its ability to induce immune response. This second one presents a better potential to find neoantigens that can be used in personalized immunotherapy. The process of neoantigen discovery typically follows a multistep pipeline that includes tumor/normal exome and transcriptome sequencing, mutation calling, human leukocyte antigen (HLA) typing, *in silico* peptide-MHC binding prediction, and immunogenicity scoring (35). Initial computational tools such as NetMHC, NetMHCpan, and MHCflurry enabled peptide binding prediction by modeling amino acid motifs and binding affinities (36, 37). More recent deep learning models, such as DeepHLApan (38) and pTuneos (39), extend beyond binding affinity to incorporate antigen processing, transcript expression levels, and peptide immunogenicity using large training datasets from immunopeptidomics and immune assays. Novel artificial intelligence (AI)-based models are being developed to integrate RNA-seq data, immunoproteasome cleavage predictions, and patient-specific immune repertoire data to better predict clinically relevant neoepitopes. Recent initiatives like the TESLA consortium have sought to benchmark prediction tools and define the most biologically relevant features of immunogenic neoepitopes (40). Despite these advances, several major translational challenges remain. The high false-positive rate in computational predictions often leads to the selection of peptides that bind MHC but are not naturally processed or presented. Additionally, tumor heterogeneity, low expression of mutant transcripts, immune evasion mechanisms (such as HLA loss), and limited understanding of TCR recognition all hinder robust epitope selection. These limitations underscore the necessity of experimental validation through immunopeptidomics (e.g., LC-MS/MS) and T-cell assays.

### 3.3 Therapeutic mRNA formulations

Dozens of different ways of mRNA formulation and delivery were tested in preclinical and clinical studies (41). As the main aim of our review is to highlight the latest clinical trials in oncology using mRNA therapeutics and broadly discuss their published results, in line with this objective, we will focus on the direct administration of mRNA therapeutics and will not delve into the results of studies involving DCs loaded with tumor antigen-encoding mRNA, which are thoroughly discussed in other review articles (42–44).

Over the past few decades, research findings on the use of naked and protamine-covered mRNA therapeutics have been published and will be discussed below. In current clinical studies, the most prevalent forms of mRNA therapeutics are LNPs and lipoplexes, as outlined in **Figure 1**.

Naked mRNA therapeutics are composed of mRNA particles diluted in a solution buffer (45) without any additional protective measures. Lack of protection makes mRNA vulnerable to enzymatic degradation upon administration into the human body. Recognizing this vulnerability, subsequent clinical studies have employed carrier-based delivery technologies.

Protamine is a cationic peptide that prevents mRNA from degradation (46) and has been evaluated in clinical trials, as discussed below. It is important to note that protamine may elicit an innate immune response and can be reactogenic. As a result, efforts have been ongoing to explore alternative carrier-based formulations for mRNA therapeutics (47).

**Figure 5:**
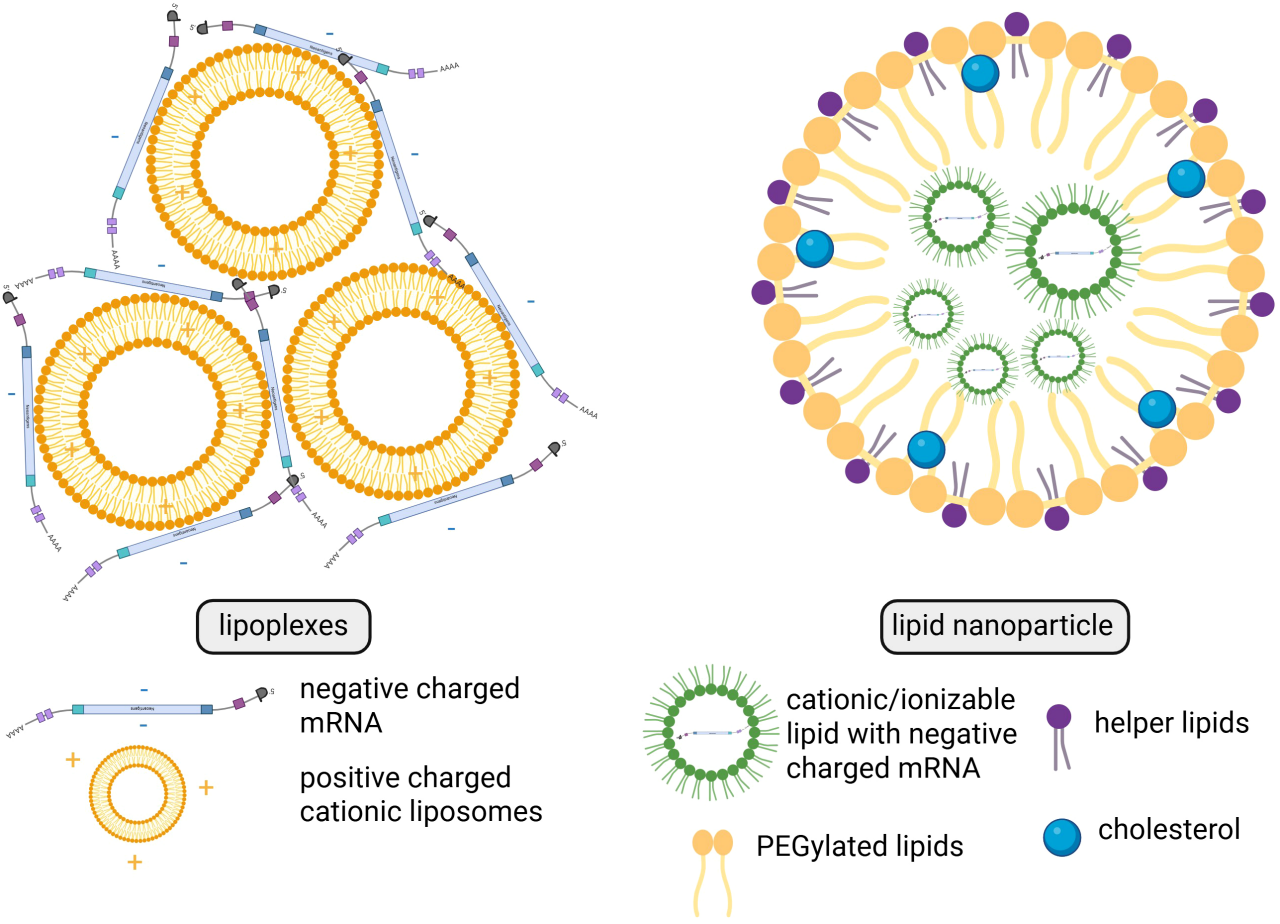
Structural and compositional differences between lipoplexes and lipid nanoparticles. Created in BioRender. Gawalska, A. (2025) https://BioRender.com/tehg8k0.

The most popular formulations being utilized in present-day clinical trials are lipoplexes and LNPs (**Figure 5**). Lipoplexes are formed by the interaction of cationic liposomes with the negative charges found on mRNA. LNPs are composed of cationic/ionizable lipid, helper lipids, cholesterol, and/or PEGylated (connected with polyethylene glycol (PEG)) lipids, which encapsulate the polyanionic mRNA and create a three-dimensional structure. LNPs not only protect mRNA from enzymatic degradation but also enhance its delivery into human cells (48).

### 3.4 Routes of administration

In clinical trials involving mRNA therapeutics, various administration routes have been explored (**Figure 1**). In earlier studies, scientists administered naked mRNA intranodally to directly target immune cells in the lymph nodes. Currently, the most utilized methods of therapeutic administration involve intramuscular and intravenous delivery of mRNA therapeutics encapsulated in lipoplexes or LNPs. Other routes investigated involve intradermal and subcutaneous delivery.

## 4 Clinical trials

At present, there are numerous ongoing clinical trials utilizing mRNA therapeutics for cancer immunotherapy. These trials are summarized in **Table 1** based on the data available on: https://www.clinicaltrials.gov (49), https://www.clin.larvol.com (50), and cancer.gov (51). Importantly, in the last 2 years many new clinical trials have been launched. Unfortunately, not all data regarding the type of formulation or route of administration could be found yet. At this point, we decided that in **Figure 1** we include only the information which is publicly available.

**Table 1:** Clinical trials utilizing mRNA vaccines (updated or published on ClinicalTrials.gov from 2016 to 2025). Data collection was completed as of 31 May 2025.

Trial number	Initiation date	Trial phase (if applicable)	Target antigens (if known)	Type of malignancy	Combination therapy (if applicable)	Formulation type	Route of administration	Sponsor	Trial status
NCT02316457	2016	Phase 1	Personalized tumor antigens with p53 RNA	Breast cancer	None	Lipoplex	Intravenous	BioNTech SE	Completed
NCT03418480	2017	Phase 1/2	Human papillomavirus type 16 (HPV-16) oncoproteins E6 and E7	Head and neck HPV16+ cancers	n/a	Lipoplex	Intradermal	University of Southampton	Completed
NCT03815058	2019	Phase 2	Personalized tumor antigens	Melanoma	Pembrolizumab (anti-programmed death receptor 1 ligand (PD-1) antibody)	Lipoplex	Intravenous	Genentech, Inc.	Completed
NCT03908671	2019	n/a	Personalized tumor antigens	Non-small cell lung carcinoma (NSCLC) and esophageal cancer	n/a	Unknown	Subcutaneous	Stemirna Therapeutics	Recruiting
NCT03948763	2019	Phase 1	Kirsten rat sarcoma virus (KRAS) gene mutations (G12D, G12V, G13D, and G12C)	KRAS mutant NSCLC, colorectal cancer or pancreatic adenocarcinoma	None or pembrolizumab	Lipid nanoparticles	Intramuscular	Merck Sharp & Dohme LLC	Terminated
NCT04163094	2019	Phase 1	Three tumor antigens	Ovarian cancer	Carboplatin/paclitaxel	Lipoplex	Intravenous	University Medical Center Groningen	Terminated
NCT04382898	2019	Phase 1/2	Five tumor antigens	Prostate cancer	None or cemiplimab (anti-PD-1 antibody)	Lipoplex	Intravenous	BioNTech SE	Terminated
NCT04486378	2021	Phase 2	Personalized tumor antigens	Colorectal cancer	n/a	Lipoplex	Intravenous	BioNTech SE	Recruiting
NCT04526899	2021	Phase 2	Cancer-testis antigen New York esophageal squamous cell carcinoma 1 (NY-ESO-1), MAGE-A3, tyrosinase, and putative tyrosine-protein phosphatase (TPTE)	Melanoma	Cemiplimab	Lipoplex	Intravenous	BioNTech SE	Active, not recruiting
NCT04534205	2021	Phase 2/3	HPV-16 oncoproteins E6 and E7	Head and neck cancer (HPV16+ and expressing PD-L1)	Pembrolizumab	Lipoplex	Intravenous	BioNTech SE	Recruiting
NCT04573140	2021	Phase 1/2	pp65 full-length (fl) lysosomal associated membrane protein (LAMP) and tumor mRNA	Pediatric high-grade gliomas and adult glioblastoma	n/a	Lipid nanoparticles	Intravenous	University of Florida	Recruiting
NCT04683939	2022	Phase 1/2	Claudin (CLDN) 18.2	Gastric, pancreatic, ovarian, and biliary tract tumors (CLDN18.2+)	None or nab-paclitaxel and gemcitabine	Lipid nanoparticles	Intravenous	BioNTech SE	Terminated
NCT05142189	2022	Phase 1	Six tumor antigens	NSCLC	None or cemiplimab or docetaxel or cemiplimab, docetaxel and carboplatin+paclitaxel or anti-cytotoxic T-cell antigen 4 (CTLA-4) antibody	Lipoplex	Intravenous	BioNTech SE	Recruiting
NCT05192460	2022	n/a	Personalized tumor antigens	Gastric cancer, esophageal cancer, and liver cancer	None or anti-PD-1/L1 antibody	Unknown	Unknown	Jianming Xu	Recruiting
NCT05198752	2022	Phase 1	Personalized tumor antigens	Solid tumors	n/a	Unknown	Subcutaneous	Stemirna therapeutics	Unknown status
NCT05202561	2022	Phase 1	KRAS gene mutation (G12C, G12D, or G12V)	Solid tumors	None or nivolumab (anti-PD-1 antibody)	Unknown	Intramuscular	First Affiliated Hospital Bengbu Medical College	Unknown status
NCT05227378	2022	n/a	Personalized tumor antigens	Gastric cancer	None or anti-PD-1/L1 antibody	Unknown	Intradermal	Shen Lin	Not yet recruiting
NCT05359354	2022	n/a	Personalized tumor antigens	Solid tumors	None or anti-PD-1 antibody	Unknown	Unknown	YueJuan Cheng	Recruiting
NCT05557591	2023	Phase 2	Six tumor antigens	NSCLC	Cemiplimab	Lipoplex	Intravenous	Regeneron Pharmaceuticals	Recruiting
NCT05660408	2025	Phase 1/2	pp65 fl LAMP and tumor mRNA	Osteosarcoma and pediatric high-grade gliomas	n/a	Lipid nanoparticles	Unknown	University of Florida	Recruiting
NCT05714748	2022	Phase 1	Epstein–Barr virus (EBV) antigen	Nasopharyngeal carcinoma (EBV+)	n/a	Unknown	Intramuscular	West China Hospital	Unknown status
NCT05738447	2023	Phase 1	Hepatitis B virus (HBV) antigen	Hepatocellular carcinoma (HBV+)	n/a	Unknown	Intramuscular	West China Hospital	Unknown status
NCT05761717	2023	n/a	Personalized tumor antigens	Liver cancer	Sintilimab (anti-PD-1 antibody)	Unknown	Subcutaneous	Shanghai Zhongshan Hospital	Not yet recruiting
NCT05916248	2023	Phase 1	Personalized tumor antigens	Solid tumors	None or pembrolizumab	Unknown	Unknown	Ruijin Hospital	Recruiting
NCT05916261	2023	Early Phase 1	Personalized tumor antigens	Pancreatic cancer	None or pembrolizumab	Unknown	Unknown	Ruijin Hospital	Recruiting
NCT05933577	2023	Phase 3	Personalized tumor antigens	Melanoma	Pembrolizumab	Lipid nanoparticles	Intramuscular	Merck Sharp & Dohme LLC	Active, not recruiting
NCT05938387	2023	Phase 1	Eight epitopes from tumor antigens	Glioblastoma or astrocytoma	n/a	Lipid nanoparticles	Intramuscular	CureVac	Active, not recruiting
NCT05940181	2023	n/a	Unknown	Solid tumors	Sintilimab	Unknown	Unknown	Jianming Xu	Recruiting
NCT05942378	2023	Phase 1	Unknown	Solid tumors	Adebrelimab (anti-PD-L1 antibody)	Unknown	Unknown	Fudan University	Not yet recruiting
NCT05949775	2023	n/a	Personalized tumor antigens	Solid tumors	Sintilimab	Unknown	Subcutaneous	Stemirna Therapeutics	Not yet recruiting
NCT05968326	2023	Phase 2	Personalized tumor antigens	Pancreatic cancer	Atezolizumab and modified leucovorin, 5-fluorouracil, irinotecan, and oxaliplatin	Lipoplex	Intravenous	Genentech, Inc.	Recruiting
NCT05981066	2023	n/a	Unknown	Hepatocellular carcinoma	n/a	Unknown	Intramuscular	Peking Union Medical College Hospital	Recruiting
NCT06019702	2023	Phase 1	Personalized tumor antigens	Digestive system neoplasms	None	Unknown	Subcutaneous	Sir Run Run Shaw Hospital	Recruiting
NCT06026800	2023	Phase 1	Personalized tumor antigens	Digestive system neoplasms	Standard first-line treatment	Unknown	Subcutaneous	Sir Run Run Shaw Hospital	Recruiting
NCT06026774	2023	Phase 1	Personalized tumor antigens	Digestive system neoplasms	Standard adjuvant therapy	Unknown	Subcutaneous	Sir Run Run Shaw Hospital	Recruiting
NCT06077760	2023	Phase 3	Personalized tumor antigens	NSCLC	Pembrolizumab	Lipid nanoparticles	Intramuscular	Merck Sharp & Dohme LLC	Recruiting
NCT06141369	2024	n/a	Personalized tumor antigens	Endocrine tumor	n/a	Unknown	Intramuscular	Shanghai Jiao Tong University School of Medicine	Recruiting
NCT06156267	2024	Early Phase 1	Personalized tumor antigens	Pancreatic cancer	Adebrelimab	Unknown	Unknown	Fudan University	Not yet recruiting
NCT06195384	2024	Phase 1	Personalized tumor antigens	Solid tumors	n/a	Unknown	Unknown	Second Affiliated Hospital of Guangzhou Medical University	Recruiting
NCT06273553	2024	Phase 1/2	HPV-16 and HPV-18 antigens	Cervical intraepithelial neoplasia	n/a	Unknown	Intramuscular	RinuaGene Biotechnology Co., Ltd.	Not yet recruiting
NCT06305767	2024	Phase 1/2	Personalized tumor antigens	Bladder cancer	Pembrolizumab	Lipid nanoparticles	Intramuscular	Merck Sharp & Dohme LLC	Recruiting
NCT06326736	2024	Early Phase 1	Personalized tumor antigens	Pancreatic cancer	Camrelizumab (anti-PD-1 antibody), gemcitabine, and abraxane	Unknown	Unknown	Jinling Hospital, China	Recruiting
NCT06353646	2024	n/a	Personalized tumor antigens	Pancreatic cancer	Ipilimumab, gemcitabine and capecitabine	Unknown	Unknown	Wu Wenming	Not yet recruiting
NCT06389591	2024	Phase 1	pp65, personalized tumor mRNA, pp65 fl LAMP mRNA	Glioblastoma	n/a	Lipoplex	Intravenous	University of Florida	Recruiting
NCT06496373	2024	Phase 1	Personalized tumor antigens	Pancreatic cancer	Anti-PD-1 antibody	Unknown	Unknown	Ruijin Hospital	Recruiting
NCT06497010	2024	Early Phase 1	Personalized tumor antigens	Solid tumors	Anti-PD-1 antibody	Unknown	Intramuscular	The Affiliated Hospital of Guizhou Medical University	Recruiting
NCT06577532	2024	Early Phase 1	KRAS mutations antigens	pancreatic cancer	None or toripalimab (anti-PD-1 antibody)	Unknown	Intramuscular	Ruijin Hospital	Recruiting
NCT06610227	2024	Early Phase 1	MHC class I polypeptide–related sequence A/B (MICA/B)	Solid tumors	n/a	Unknown	Intramuscular	NING LI	Not yet recruiting
NCT06685653	unknown	Early Phase 1	Personalized tumor antigens	NSCLC	Adebrelimab	Unknown	Unknown	Nanjing Tianyinshan Hospital	Not yet recruiting
NCT06735508	2025	Early Phase 1	Personalized tumor antigens	NSCLC	Adebrelimab	Unknown	Unknown	Guangdong Provincial People’s Hospital	Not yet recruiting
NCT06741150	2024	n/a	HPV-16 antigen	Cervical, vaginal, and vulvar intraepithelial neoplasia and cancer (HPV-16+)	n/a	Unknown	Intramuscular	Newish Technology (Beijing) Co., Ltd.	Recruiting
NCT06788600	2025	Unknown	EBV antigen	Lymphoma (EBV+)	None	Unknown	Unknown	Ruijin Hospital	Not yet recruiting
NCT06833073	2025	Phase 2	Personalized tumor antigens	Bladder cancer	Bacillus Calmette-Guerin vaccine	Lipid nanoparticles	Intramuscular	Merck Sharp & Dohme LLC	Recruiting

In our review, we focus and discuss in detail clinical trials with already published results. Data summarizing these trials are presented in **Table 2**. The trials were classified and discussed accordingly to the type of cancer targeted by the vaccine. Very importantly, mRNA vaccines have mostly been tested in phases 1 and 2; however, some therapeutics with most promising results from early phases have already entered ongoing phase 3 trials.

**Table 2:** Outcomes of clinical trials with published results (updated or published on ClinicalTrials.gov from 2004 to 2025).

Trial number	Initiation date	Trial phase (if applicable)	Target antigens (if known)	Type of malignancy	Combination therapy (if applicable)	Formulation type	Route of administration	Sponsor	Trial status	Additional citation (if applicable)
NCT00204516	2007	Phase 1/2	Melan-A, Mage-A1, Mage-A3, Survivin, Glycoprotein 100 (GP100), and tyrosinase or personalized tumor antigens	Melanoma	Granulocyte-macrophage colony-stimulating factor (GM-CSF)	Naked	Intradermal	University Hospital Tuebingen	Completed	(29)
NCT00204607	2004	Phase 1/2	Melan-A, Mage-A1, Mage-A3, Survivin, GP100 and Tyrosinase	Melanoma	GM-CSF	Protamine	Intradermal	University Hospital Tuebingen	Completed	(52)
NCT00831467	2009	Phase 1/2	Prostate-specific antigen (PSA), prostate-specific membrane antigen (PSMA), prostate stem cell antigen (PSCA), and six-transmembrane epithelial antigen of the prostate (STEAP)	Prostate cancer	n/a	Protamine	Intradermal	CureVac	Completed	(53)
NCT00906243	2009	Phase 1/2	PSA, PSMA, PSCA and STEAP	Prostate cancer	n/a	Protamine	Intradermal	University of Florida	Terminated	(54) (55)
NCT00923312	2009	Phase 1/2	NY-ESO-1, MAGE-C1/CT7, MAGE-C2/CT10, survivin, and trophoblast glycoprotein (5T4)	NSCLC	n/a	Protamine	Intradermal	CureVac	Completed	(54) (56)
NCT01684241	2012	Phase 1	2 TAAs	Melanoma	n/a	Naked	Intranodal	BioNTech SE	Completed	n/a
NCT01817738	2012	Phase 1/2	PSA, PSMA, PSCA, STEAP1, PAP, and MUC1	Prostate cancer	n/a	Protamine	Intradermal	CureVac	Terminated	(57)
NCT01915524	2013	Phase 1	NY-ESO-1, MAGE-C1, MAGE-C2, survivin, 5T4, and MUC-1	NSCLC	Local radiation, pemetrexed, and (epidermal growth factor receptor tyrosine kinase inhibitor) EGFR-TKI	Protamine	Intradermal	CureVac	Terminated	(58) (59)
NCT02035956	2013	Phase 1	Personalized tumor antigens	Melanoma	n/a	Naked	Intranodal	BioNTech RNA Pharmaceuticals GmbH	Completed	(45) (60)
NCT02140138	2014	Phase 2	PSA, PSMA, PSCA, STEAP1, prostatic acid phosphatase (PAP), and mucin-1 (MUC1)	Prostate cancer	n/a	Protamine	Intradermal	CureVac	Terminated	(57)
NCT02410733	2015	Phase 1	NY-ESO-1, tyrosinase, MAGE-A3, and TPTE	Melanoma	None or anti-PD-1 antibody	Lipoplex	Intravenous	BioNTech SE	Completed	(61)
NCT03164772	2017	Phase 1/2	MUC1, survivin, NY-ESO-1, 5T4, MAGE-C2, and MAGE-C1	NSCLC	Durvalumab or durvalumab and tremelimumab	Protamine	Intradermal	Ludwig Institute for Cancer Research	Completed	(62)
NCT03289962	2017	Phase 1	Personalized tumor antigens	Solid tumors	Atezolizumab (anti-PD-L1 antibody)	Lipoplex	Intravenous	Genentech	Completed	(63)
NCT03313778	2017	Phase 1	Personalized tumor antigens	Solid tumors	None or pembrolizumab or SoC treatment or pembrolizumab and SoC treatment	Lipid nanoparticles	Intramuscular	ModernaTX, Inc.	Recruiting	(64)
NCT03394937	2017	Phase 1	TriMix (mRNAs encoding cluster of differentiation 40 ligand (CD40L), CD70 and caTLR4), and tyrosinase, gp100, MAGE-A3, MAGE-C2, and preferentially expressed antigen of melanoma (PRAME)	Melanoma	None	Naked	Intranodal	eTheRNA immunotherapies	Terminated	(65)
NCT03468244	2018	n/a	Personalized tumor antigens	Digestive system neoplasms	None	Unknown	Subcutaneous	Changhai Hospital	Unknown status	(66)
NCT03480152	2018	Phase 1/2	Up to 20 personalized tumor antigens	Gastrointestinal cancer	None	Lipid nanoparticles	Intramuscular	National Cancer Institute (NCI)	Terminated	(67)
NCT03897881	2019	Phase 2	Personalized tumor antigens	Melanoma	Pembrolizumab	Lipid nanoparticles	Intramuscular	ModernaTX, Inc.	Recruiting	(68)
NCT04161755	2019	Phase 1	Personalized tumor antigens	Pancreatic cancer	Atezolizumab and mFOLFIRINOX	Lipoplex	Intravenous	Memorial Sloan Kettering Cancer Center	Active, not recruiting	(69)
NCT04503278	2020	Phase 1	CLDN6	Solid tumors (CLDN6+)	CLDN6 CAR-T	Lipoplex	Intravenous	BioNTech Cell & Gene Therapies GmbH	Recruiting	(70)

### 4.1 Melanoma

mRNA formulations against malignant melanoma were one of the first tested in clinical trials. This type of skin malignancy still poses a major mortality rates worldwide and due to its one of the highest tumor mutation burdens (TMB) (71) becomes a perfect target for the application of mRNA vaccines.

One of the earliest trials (NCT00204516) was published in 2008 by Weide et al. (29). Their study included 15 patients—6 with stage III and 9 with stage IV melanoma. All participants had advanced cancer with metastases, which was a key inclusion criterion for this study. The investigators analyzed frozen tumor samples and used the material to create autologous mRNA libraries for each patient. Subsequently, the naked mRNA therapeutic (**Table 2**) was injected intradermally in combination with granulocyte-macrophage colony-stimulating factor (GM-CSF) whose role was to enhance the immune response by promoting DC activation and function. The vaccine was well-tolerated, with no grade 3 or 4 toxicity observed. Additionally, it successfully triggered antibody production and T-cell activation in a subset of patients. However, the small sample size did not allow to draw definitive conclusions about the clinical effectiveness of this treatment.

Weide et al. further investigated the potential of mRNA vaccines in patients with metastatic melanoma (52). Unlike the initial study, this trial (NCT00204607) focused on defined melanoma antigens. The vaccine targeted various antigens including those specific and crucial for the function of melanocytes such as Melan-A, gp100, and tyrosinase. However, other antigens Mage-A1, Mage-A3, and Survivin are not unique to melanoma but have a well-established role in the pathogenesis of melanoma. Additionally, the mRNA was further stabilized with protamine, and its administration followed a more intensified schedule at higher doses. As in the first trial, GM-CSF was used as an additional therapy. However, patients were randomized into two groups, with one group also receiving keyhole limpet hemocyanin (KLH), a substance previously demonstrated to be a necessary complement to GM-CSF (72). The study enrolled 21 patients with stage III or IV melanoma, 10 of whom completed the entire protocol, which consisted of 12 intradermal vaccinations administered over 19 weeks. The vaccine was shown to be safe, with no serious side effects reported, although the inclusion of protamine resulted in more pronounced injection site reactions. The research demonstrated that the vaccine could significantly decrease Foxp3^+^/CD4^+^ regulatory T cells in the peripheral blood of patients in the KLH arm, whereas in the non-KLH group, the subset of myeloid suppressor cells (CD11b^+^HLA-DR^lo^ monocytes) was reduced. These immunological effects may further enhance the immune response to cancer cells, potentially improving outcomes for melanoma patients. Although the study did not specifically assess clinical outcomes, the participants were followed for up to 36 months after enrollment. One patient with lung metastases achieved a partial response following the full vaccination cycle that was followed by additional vaccinations and the treatment course eventually resulted in complete remission. 16 months after the trial began, the patient developed bone metastasis, which was surgically removed. Remarkably, no signs of relapse were observed until the end of the observation period. While the study findings were promising, they were limited by the small sample size.

In 2017, Sahin et al. reported the first-in-human application of personalized mRNA vaccine in melanoma (45, 60). This study (NCT02035956) tested naked mRNA vaccine (**Table 2**) administered intranodally to 13 patients diagnosed with stage III or IV melanoma with a recent history of recurrence and high risk of relapse. All patients received individually prepared vaccine targeting 10 selected neoepitopes. T-cell responses against most of them were detected in all patients, and vaccines were well-tolerated without any serious adverse effect. From the selected group of 13 patients, 8 patients had no radiologically detectable lesions at the beginning of the trial and this group exerted the strongest response to the vaccination and remained recurrence-free the whole follow-up period (12 to 23 months). However, 5 patients have relapsed soon after inclusion and had progressing metastases at the point of vaccination. From this group, one patient has achieved a complete response (CR) and another one had a vaccine-related partial response (PR).

What is needed to emphasize is that there were only few studies investigating naked mRNA vaccines. This type of formulation is easily degraded by RNAses, making it highly susceptible to the environment. In the other one study (NCT03394937), 20 patients with stage IIc/III/IV resected melanoma received five administrations of intranodal ECI-006 in combination with standard ICI treatment. The therapeutic was a combination of TriMix and mRNAs encoding five TAAs. TriMix is an mRNA formulation encoding cluster of differentiation 40 ligand (CD40L), CD70, and caTLR4. It works as a booster promoting the maturation and activation of dendritic cells. The treatment did not provoke any significant side effects and was well-tolerated among patients. The immunogenic effect was exerted in the part of the tested group (65).

Another breakthrough study investigating mRNA technology in melanoma from Sahin and colleagues was published in 2020. They demonstrated the results from the phase 1 Lipo-MERIT trial (NCT02410733). The tested was FixVac (BNT111)—a liposomal-mRNA vaccine (**Table 2**) which targeted four TAAs common in melanoma. The safety of the formulation was tested among 89 patients with stage IIIB/IIIC and IV melanoma—no severe adverse effects or dose limiting toxicity was reported. The efficacy of the drug was analyzed in the group of 42 patients with measurable metastatic disease. A total of 25 patients received only FixVac: 3 patients experienced PR, 7 stable disease (SD), and 1 a CR of the disease. Other 17 patients were given the combination of FixVac with anti-PD-1 treatment, and 7 patients from this group developed a partial response. Importantly, treatment with FixVac has promoted the expansion and activation of tumor-specific T cells, especially in patients with PR (61). The promising results from this study have paved the route for launching the phase 2 trial (NCT04526899).

Finally, in the Keynote-942 clinical trial (NCT03897881) with individualized neoantigen mRNA vaccine mRNA-4157/V940 (**Table 2**), 157 patients were randomized to two groups: one treated with the combination of mRNA-4157/V940 with pembrolizumab (n=107) and the second treated with pembrolizumab in monotherapy (n=50). The study has demonstrated significant improvement in remission-free survival (RFS) in the combination therapy compared with monotherapy (the 18-month RFS rates were 79% vs. 62%, respectively), the reduction of the risk of recurrence or death by 22% vs. 40% and prolonged distant metastasis-free survival (DMFS). Promising results from this study led to the decision to initiate in 2023 the phase 3 trials in patients with advanced melanoma (NCT05933577) and NSCLC (NCT06077760) (68).

### 4.2 Non-small cell lung cancer

Another cancer, non-small cell lung cancer (NSCLC), remains a major therapeutic challenge, which prompts search for a new therapeutic strategy. The positive results from the use of ICIs such as in the case of melanoma have encouraged further research for the application of mRNA vaccines in the treatment of this cancer.

Notably, in 2019, the first clinical application (NCT00923312) of CV9201 (RNActive^®^ antigen-specific therapeutic) (**Table 2**) designed to target five NSCLC TAAs (56), developed by CureVac, yielded encouraging findings. In a phase I/IIa multicenter, open-label, uncontrolled trial, the primary objective was to assess the safety of the vaccine and evaluating impact on immune response induction. The study enrolled 46 patients with stage IIIB/IV NSCLC following first-line treatment with either chemotherapy or chemoradiotherapy. The phase I objective was to determine the recommended phase 2 dose, whereas the phase IIa extension investigators focused on evaluating vaccine safety and immune response. The dose-limiting toxicity (DLT) was not observed in any of the tested groups, allowing the highest dose to be tested in a cohort of 37 patients during phase IIa. The vaccine was well-tolerated, with 85% of adverse events (AEs) classified as grade 1. Immune responses were detected in 63% of patients, with antigen-specific immune responses observed against at least one of the targeted antigens. Although no objective tumor response was noted, it is well established that cancer vaccines alone are unlikely to elicit significant clinical responses in advanced-stage diseases. Consequently, the study authors recommended further investigation of CV9201 in combination with other active therapies, including ICIs.

The promising results from the previously trial have paved the way for further evaluation (NCT01915524) of the CV9202 (**Table 2**) protamine vaccine (58). This therapeutic targeted the same five antigens as the earlier trial with additional the mucin-1 (MUC-1) antigen. The purpose of the trial was to assess the potential of combining this vaccine with radiotherapy, a well-established therapeutic option for NSCLC. The study enrolled 26 patients with stage IV NSCLC who had either a PR or an SD after first-line treatment. These patients were subsequently divided into three study arms (strata) based on the cancer subtype. Each stratum received intradermal administration of the vaccine along with 20 Gy of radiation therapy, and part of patients depending on cancer subtype received additional therapy (pemetrexed or epidermal growth factor receptor tyrosine kinase inhibitor (EGFR-TKI)). The primary endpoint of the trial was to evaluate the safety and tolerability of the vaccine, with the predefined margin of ≤30% of patients experiencing AEs of grade 3 or higher. The frequencies of AEs in most patients were well below this margin, with only 12.5% of patients in each group reporting grade 3 or higher AEs. Secondary endpoints included the assessment of both cellular and humoral immune responses. Of the 25 patients included in the immunological evaluation, 84% exhibited at least a twofold increase in immune response to at least one of the targeted antigens. Notably, approximately half of the patients demonstrated an increase in T-cell or antibody responses against more than one antigen. What is worth mentioning is that a subset of patients also showed immune responses against other tumor antigens that were not targeted by the vaccine. These findings suggest a potential synergistic effect between the vaccine and radiation therapy. The trial also highlighted an important consideration regarding the efficacy of mRNA vaccines: the route of administration. The use of a needle-free injection device was found to be superior to traditional needle-and-syringe injections in inducing antibody production.

The same vaccine was tested in a separate trial (NCT03164772) with combined therapy with ICIs—durvalumab and with or without tremelimumab. This phase 1b trial included 57 patients with metastatic NSCLC, who were randomized into two study arms: one group received the vaccine and durvalumab, whereas the other received a triple combination of the vaccine, durvalumab, and tremelimumab. The treatment regimen was well-tolerated, with no serious adverse events observed in any of the groups. The study secondary endpoint aimed to assess the potential efficacy of the combination therapies. The overall response rate (ORR) in the first group was 29%, whereas the second group had an ORR of 11%. The addition of the vaccine to durvalumab enhanced the treatment response rates compared with monotherapy. However, the inclusion of tremelimumab did not result in further improvement (62, 73).

Finally, individualized neoantigen mRNA vaccine mRNA-4157/V940 (**Table 2**) developed by Moderna was tested in a phase 1 clinical trial (KEYNOTE-603, NCT03313778) in patients with different types of resectable solid tumors (NSCLC and bladder cancer). Among 33 patients, 13 received vaccine in monotherapy and 20 in combination with pembrolizumab. The treatment was safe and induced the production of tumor-specific immune response (64).

### 4.3 Genitourinary cancers

The search for new treatment options to improve survival rates in patients with advanced castration-resistant prostate cancer (CRPC) also remains ongoing. Immunotherapies, such as Sipuleucel-T, have shown potential efficacy (74), and mRNA vaccines are also promising candidates in this area.

One such example is CV9103, an RNActive^®^ vaccine developed by CureVac, which targets four TAAs (**Table 2**). In a Phase I/IIa trial (NCT00831467), 44 patients with CRPC were enrolled. While 89% of patients experienced AEs, most were of mild to moderate. The vaccine also elicited an objective immunological response in 26 of 33 evaluable patients, with 58% of responders showing a response to more than one antigen. To evaluate clinical efficacy, investigators measured prostate-specific antigen–progression-free survival (PSA-PFS), which was calculated as PSA serum level progression from the beginning of vaccination. The median PSA-PFS was 1.8 months (95% CI: 1.4–3.2), and the 6-month PSA-PFS rate was 15.9%. One patient achieved a confirmed PSA response (53).

Following the previous trial, CV9104—an updated version of the earlier therapeutic—was assessed. In addition to targeting the same four antigens, CV9104 also included the MUC1 antigen. The objective of this trial (NCT01817738) was to evaluate the efficacy of the vaccine in combination with standard-of-care treatment, compared with a placebo. A total of 197 patients with chemo-naïve, oligosymptomatic/asymptomatic metastatic CRPC without visceral metastases were randomly assigned to receive either the vaccine (n=134) or placebo (n=63), in addition to standard treatment. The primary endpoint, overall survival (OS), showed no significant difference between the two groups, with OS of 35.5 months in the vaccine group compared with 33.7 months in the placebo group. Investigators also assessed radiographic PFS, but again, no significant differences were found between the groups (57).

Due to the successful application of BCG (Bacillus Calmette–Guerin) vaccine in the treatment regimen for bladder cancer according to various current guidelines (75, 76), the search for other forms of immunotherapy is ongoing. As mentioned previously, Moderna has already demonstrated promising results with its vaccine in the KEYNOTE-603 trial (NCT03313778) (64) that included also a subgroup of patients with bladder cancer. The same formulation intismeran autogene (refereed also as mRNA 4157) is now evaluated in two ongoing trials. The Phase II INTerpath-011 trial (NCT06833073) is actively recruiting patients with high-risk non-muscle invasive bladder cancer (NMIBC) to receive mRNA-4157 in combination with standard BCG therapy. In parallel, the Phase I/II INTerpath-005 trial (NCT06305767) is investigating the efficacy of this therapeutic approach alongside pembrolizumab, with or without enfortumab vedotin, in muscle-invasive disease. Completion of these trials is projected for 2031, potentially paving the way for a new class of personalized immunotherapies in urothelial carcinoma.

### 4.4 Other solid tumors

The technology of mRNA vaccines has also been evaluated in patients with gastrointestinal neoplasms. A significant breakthrough from the phase 1 clinical trial (NCT04161755) of cevumeran was published in May 2023 (69). This mRNA vaccine, which encodes personalized neoantigens and is delivered in a lipoplex nanoparticle formulation, was tested in patients with pancreatic ductal adenocarcinoma (PDAC). PDAC remains one of the most challenging cancers, characterized by high mortality rates and late-stage diagnosis (77). This may be partly due to the limited number of mutations in PDAC, which results in a scarcity of neoantigens (78), and a corresponding weak immune response. However, cevumeran has shown promise as a potential therapeutic option for these patients. In the study, 28 out of 34 enrolled patients underwent surgical resection of their tumors. These tumors were subsequently analyzed for mutations, and a personalized vaccine was developed and administered to 16 of the patients with combination therapy (**Table 2**). The results demonstrated that the vaccine was able to trigger a T-cell immune response against at least one of the neoantigens in half of the patients. Remarkably, half of the responders developed a response to multiple antigens. At the 18-month median follow-up, all patients in the responder group had a longer median RFS (not yet reached, with all patients still alive) compared with non-responders, who had a median RFS of 13.4 months (69).

The same formulation was also tested in another phase 1 trial (NCT03289962) in a group of patients with advanced, metastatic, or recurrent malignancies including colorectal, bladder, NSCLC, melanoma, and renal cell carcinoma. Patients included in the study received either cevumeran in monotherapy or in combination with atezolizumab. The vaccine has proven to be well-tolerated among the studied group, eliciting mostly mild treatment-related adverse effects, mostly infusion-related reactions. The formulation induced poly-epitopic neoantigen-specific responses in 71% of patients, which was not detectable at baseline (63).

A study from China (NCT03468244) investigated the use of a personalized mRNA vaccine in patients with advanced rectal, colon, and gastric cancers. The study involved only three patients, each with a different type of gastrointestinal cancer. The tested vaccine combination was found to be safe, with no serious AEs reported. Moreover, it successfully activated an immune response, significantly increasing circulating interleukin levels (66).

Similarly, Moderna explored the potential of its mRNA vaccine in trial NCT03480152 treating four patients with advanced metastatic gastrointestinal cancers: one with gastric cancer, two with rectal cancer, and one with colon cancer. These patients had already undergone extensive treatments, including ICIs and tumor-infiltrating lymphocyte (TIL) therapy. The intramuscular mRNA-4650 therapeutic was designed to target specific neoantigens expressed by the tumor cells (**Table 2**). The vaccine was shown to be safe, with only grade 1 and 2 AEs observed in this small cohort. Although no clinical responses were noted, the presence of both CD4^+^ and CD8^+^ neoantigen-specific T-cells post-vaccination suggests immune response activation (67).

The encouraging results from the completed trials have also motivated further investigation of mRNA vaccines in gastrointestinal cancers. For instance, we are still awaiting the results from Chinese studies currently recruiting patients with hepatocellular carcinoma (NCT05761717, NCT05738447), which may open a new interesting treatment option for this group of patients.

mRNA vaccine technology has also been explored by BioNTech in combination with another groundbreaking oncology approach: chimeric antigen receptor (CAR) T cells. The updated results from the ongoing Phase 1/2 trial (NCT04503278) of the CAR-T cell-amplifying RNA vaccine (CARVac) were published in September 2024. The CAR-T cells target oncofecal antigen claudin 6 (CDLN6), which is expressed in various solid tumors (79). The role of mRNA is to boost the activity of CAR-T cells. Formulated mRNA encoding CDLN6 enters APCs cells to stimulate the expression of CDLN6 on their surface. This mechanism further activates CAR-T cell activity and promotes their expansion. The conducted study involved 59 patients, with 26 receiving CAR-T monotherapy and 33 receiving a combination of CAR-T and the mRNA vaccine. The primary endpoint of the study was to evaluate safety and tolerability, which was the main concern of this combination. Treatment-related AEs were observed in 88% of patients, with 64% experiencing Grade 3 AEs and 39% reporting serious AEs. In secondary endpoints, the ORR was 38% (20 of 58 patients). Complete results from the trial are still pending (70).

## 5 Safety profile of mRNA vaccines

Given the growing number of ongoing trials exploring mRNA therapeutics in oncology and the current lack of widespread application of these among patients, it remains premature to draw definitive conclusions regarding the safety profile of mRNA vaccines in oncology. However, basing on the broad use of mRNA technology in infectious diseases and already published results from completed studies, we can remain hopeful that it can become a safe addition to standard of care in many treatment regimens. Most available data currently stem from phase 1 and 2 clinical trials, which primarily aimed to assess the safety profiles of investigational combinations.

In all studies described before (**Table 2**), all formulations have proven to be well-tolerated among patients overall. The majority of reported AEs were Grade 1 or 2, with few instances of serious side effects. Frequently observed AEs included fatigue, fever, injection-site reactions, and transient flu-like symptoms. Notably, no DLTs were reported, even at the highest administered doses across multiple trials (56, 58). The wide application of immune-checkpoint inhibitors has led to the appearance of more cases of immune-related adverse effects (irAEs), raising concerns about the potential for mRNA technologies to induce autoimmune reactions. One trial evaluating the CV9201 vaccine in NSCLC (56) also monitored antibody levels to assess this risk. Although the levels were elevated, they did not correlate with the increase of autoimmune diseases. Nevertheless, this aspect requires further investigation to fully understand the immunological impact of mRNA-based therapeutics.

## 6 Discussion about futures perspectives and obstacles

The unprecedented success that achieved mRNA vaccine during COVID-19 pandemic demonstrated their potential to the whole world. Thousands of research groups are conducting their studies to implement an mRNA platform to present vaccinology not only against infectious diseases, but also in oncology and other medicine areas (**Figure 1**). In cancer research, mRNA vaccines have shown significant promise in both preclinical and clinical studies, although many challenges remain. Researchers continue to address gaps in our understanding of tumor immunogenicity and vaccine design.

For several years, groups of researchers have been endeavoring to discover new potential targets that can effectively stimulate an immune response against tumors for a flexible and rapid platform for vaccine production—mRNA. The current trend in this area is to analyze The Cancer Genome Atlas (118) according to expression patterns of genes unique for each cancer to find tumor antigens, which can be a candidate for universal mRNA vaccine development. The findings from these studies across various types of cancers are outlined in **Table 3**. These results can assist scientists in identifying new potential targets for innovative mRNA therapeutics in the field of oncology.

**Table 3:** New promising mRNA vaccine targets. The featured targets were compiled from online open-source databases such as TCGA.

Type of malignancy	Potential target	Year of publication	Citation
Acute myeloid leukemia	CDH23, LRP1, MEFV, MYOF, and SLC9A9	2023	(80)
Bladder cancer	IGF2BP2 and MMP9	2022	(81)
	AP2S1, P3H4, and RAC3	2022	(82)
Breast cancer	CD74, IRF1, and PSME2	2022	(83)
Clear cell renal cell carcinoma	ARHGEF3	2023	(84)
	LRP2 and DOCK8	2023	(85)
	TOP2A, NCF4, FMNL1 and DOK3	2021	(86)
Colon adenocarcinoma	IGF2BP3, DPCR1, HOXD10, TRIM7, and ZIC5	2022	(87)
Endometrial carcinoma	PGR, RBPJ, PARVG and MSX1	2023	(88)
Esophageal squamous cell carcinoma	MMD, MTDH, and TRFC	2024	(89)
	NLRC5, LCP2, TMEM229B, and FCRL4	2022	(90)
Gastric adenocarcinoma	RAI14 and NREP	2022	(91)
Gastrointestinal mucosa-associated lymphoid tissue lymphoma	KLHL14	2022	(92)
Glioblastoma	ARHGAP9, ARHGAP30, CLEC7A, MAN2B1, ARPC1B and PLB1	2022	(93)
Head and neck squamous cell carcinoma	SREBF1, LUC7L3, LAMA5, PCGF3, HNRNPH1, KLC4, and OFD1	2022	(94)
	CCR4, TMCO1, and SPACA4	2022	(95)
Hepatocellular carcinoma	AURKA, CCNB1, CDC25C, CDK1, TRIP13, PES1, MCM3, PPM1G, NEK2, KIF2C, PTTG1, KPNA2, and PRC1	2023	(96)
	POLR3C and KPNA2	2023	(97)
	FXYD6, JAM2, GALNT16, C7, and CCDC146	2023	(98)
	PES1, MCM3, PPM1G, and KPNA2	2022	(99)
High-grade serous ovarian cancer	ARPC1B, ELF3, VSTM2L, and IL27RA	2023	(100)
Lower-grade glioma and glioblastoma	PTBP1, SLC39A1, MMP9 and SLC16A3	2022	(101)
Lung adenocarcinoma	CARD8, NAIP, NLRP1, and NLRP3	2024	(102)
	AGPS, NRAS, MTDH, PANX1, NOX4, and PPARD	2024	(103)
	ZC3H12D and TXNDC5	2022	(104)
	CCNB1, KIAA0101, PBK, OIP5 and PLEK2	2022	(105)
	GPRIN1, MYRF, PLXNB2, SLC9A4, TRIM29, UBA6, and XDH	2021	(106)
Lung squamous cell carcinoma	BMP5 and CLDN5	2022	(107)
Melanoma	PTPRC, SIGLEC10, CARD11, LILRB1 and ADAMDEC1	2022	(108)
Mesothelioma	FAM134B, ALDH3A2, SAV1, RORC, and FN1	2022	(109)
	AUNIP, FANCI, LASP1, PSMD8, and XPO5	2022	(110)
Pancreatic cancer	ERAP2, MET, CXCL9, and AGT	2024	(111)
	PAAD, ANO6, PAK2, CHMP2B, and RAB5A	2024	(112)
Papillary renal cell carcinoma	ALOX15B, HS3ST2, PIGR, ZMYND15 and LIMK1	2023	(113)
Prostate adenocarcinoma	FUS, LMNB2, RNPC3, and ZNF700	2024	(114)
Renal cell carcinoma	DBH-AS1	2022	(115)
Small cell lung cancer	NEK2, NOL4, RALYL, SH3GL2, and ZIC2	2023	(116)
Soft tissue sarcoma	HLTF, ITGA10, PLCG1, and TTC3	2022	(117)

While a fully universal mRNA-based cancer vaccine remains elusive due to the complexity and heterogeneity of tumors, the idea of a universal mRNA vaccine is being explored in other therapeutic areas. For instance, several research groups and companies, such as Centivax (119) and the NIH (120), are currently investigating mRNA-based candidates for a universal influenza vaccine. Preclinical studies involving mRNA constructs encoding antigens from all 20 influenza subtypes have demonstrated broad immune responses in animal models, and early-phase clinical trials are already underway. Although such efforts face their own challenges, they suggest that universal vaccination using mRNA platforms may be feasible in less antigenically diverse diseases (121).

Unfortunately, cancer cells may vary in the type of presenting antigens even in the subgroup of patients with the same type of tumor. This variability means that a fully universal mRNA cancer vaccine remains highly unlikely in the near future, although not categorically impossible. Careful qualification of such statements is necessary, as some tumors may share common antigens suitable for semi-personalized strategies.

In contrast, personalized mRNA cancer vaccines targeting neoantigens unique to an individual’s tumor have gained traction as a feasible and promising solution. Technological advances in bioinformatics, AI, and machine learning now allow for more accurate identification of immunogenic neoepitopes. Tools predicting neoantigen presentation on MHC molecules and T-cell recognition (e.g., NetMHCpan, MuPeXI) are already in use to support such personalized designs (37). Ongoing clinical trials, such as those by Moderna (mRNA-4157/V940 in combination with pembrolizumab, NCT03897881), demonstrate the translational potential of this approach and reinforce its growing clinical relevance (68).

Another important impulse for continuing development of mRNA technology was the Nobel Prize Award for Katalin Karikó and Drew Weissman in 2023. Their discovery about methylopseudouridine application in the mRNA sequence that is administered into living organisms received appreciation from the Nobel Prize Committee. On the day when these esteemed scientists received their Awards in Stockholm, the study conducted by Mulroney and colleagues casted a shadow on the mRNA technology (122). Their research demonstrated that the inclusion of methylopseudouridine in the mRNA sequence resulted in a translation flip and the emergence of an unexpected by-product. Furthermore, it was discovered that this product could potentially trigger an immune response against itself, which was demonstrated among individuals vaccinated with the SARS-CoV-2 mRNA vaccine. This observation will likely lead to increased scrutiny regarding the use of methylopseudouridine in the mRNA sequence during the production of mRNA vaccines. It is crucial to establish the safety of the protein expressed by this frameshift and ensure that it does not induce an immune response against healthy tissues and cells.

## 7 Conclusion

mRNA vaccines, as a platform to induce an effective immune response against cancer cells, hold great potential. Broad application in the area of infectious diseases is already present. In oncology, advancements in identifying neoantigens may make mRNA a crucial player in cancer immunotherapy. This is a critical future direction for mRNA therapeutics. To achieve progress, we need to still develop a robust platform for the accurate prediction and selection of neoantigen candidates. Scientists still do not fully understand which neoantigens can actually trigger strong antitumor immune responses. Understanding this is fundamental to unlocking the full potential of mRNA-based cancer vaccines. In the search for alternatives to traditional vaccine platforms, mRNA offers a wide array of advantages, including efficient activation of B and T cells, no requirement for adjuvants, standardized production processes, and adaptable, rapid manufacturing, which have positioned it as a leader in the vaccine development race. The recent publication of studies and the Nobel Prize awarded to Katalin Karikó and Drew Weissman have served as motivation for scientists and companies to intensify their efforts in the field of mRNA technology. The number and promising outcomes of clinical studies highlighted in this review demonstrate the potential of mRNA therapeutics in oncology. However, we need to recognize that mRNA-based therapy is unlikely to serve as the ultimate cure for cancer. Instead, mRNA therapies will likely need to be combined with other treatments including not only ICIs but also agents that target the key pathways used by tumors to evade the immune system. Only in combined therapy can mRNA take over a central role in effective cancer treatment. Present research should focus on better understanding of neoantigens, improving delivery systems, and designing combination strategies to fully demonstrate mRNA therapeutics’ potential in oncology.
